# Treatment of alcohol use disorder via magnetic resonance-guided focused ultrasound ablation surgery

**DOI:** 10.1186/s13722-025-00644-0

**Published:** 2026-01-14

**Authors:** Fang Wang, Halimureti Paerhati, Zhengyu Lin, Shuangbao Liu, Bomin Sun, Dianyou Li

**Affiliations:** 1 Department of Neurosurgery, Center for Functional Neurosurgery, Ruijin Hospital, Shanghai Jiao Tong University School of Medicine, Shanghai, 200025 China; 2 Clinical Research Center for Mental Disorders, Shanghai Pudong New Area Mental Health Center, School of Medicine, Tongji University, Shanghai, 200124 China; 3 Shanghai International Medical Center, Shanghai, 201315 China

**Keywords:** Magnetic resonance-guided focused ultrasound, Nucleus accumbens, Alcohol use disorder, Case report, Neuromodulation, Addiction

## Abstract

**Background:**

Alcohol use disorder (AUD) is a problematic pattern of drinking that can cause significant impairment or distress. Magnetic resonance-guided focused ultrasound (MRgFUS) is a novel noninvasive technique permitting incision-less ablative treatment, which may be effective for AUD.

**Case Presentation:**

We present the case of a 54-year-old man diagnosed with AUD for 30 years. After bilateral nucleus accumbens (NAc) MRgFUS, his craving for alcohol decreased markedly from 6 to 0, and he remained abstinent throughout the follow-up (12 months). Moreover, psychiatric and neuropsychological assessments showed improvements in depression, anxiety, sleep, and quality of life. Besides, he did not have any serious adverse effects during the whole follow-up period.

**Conclusions:**

This case report suggests the feasibility and potential of NAc ablation via MRgFUS in treating severe AUD. Subsequent multi-sample double-blind controlled trials need to be further studied. The effect of MRgFUS on the use disorders of other substances also requires further research.

**Supplementary information:**

The online version contains supplementary material available at 10.1186/s13722-025-00644-0.

## Background

Alcohol Use Disorder (AUD) is a medical condition characterized by impaired control over alcohol consumption, compulsive drinking behaviors, and persistent use despite adverse health, occupational, or social consequences [1]. AUD is a prevalent global health challenge. According to the World Health Organization’s latest 2024 report, an estimated 400 million people (7% of the global adult population) were living with AUD in 2019 [2]. In the same year, alcohol consumption was responsible for 2.6 million deaths worldwide, accounting for 4.7% of all global mortality. Despite some progress, this immense disease burden, further exacerbated by a global treatment gap that leaves over half of all people with AUD untreated (particularly in low- and middle-income countries), underscores the urgent need for novel and effective interventions [2]. A 2019 national survey estimated that 4.4% of Chinese adults suffer from AUD lifetime [3]. In addition to multiple psychosocial and pharmacologic treatments, neuromodulation therapies have shown some effects in the treatment of AUD, such as repeated transcranial magnetic stimulation (rTMS) and deep brain stimulation (DBS). rTMS is noninvasive, but unable to target subcortical regions such as the nucleus accumbens (NAc), which is a central hub in the circuitry of addiction. DBS can precisely target both cortical and subcortical areas, but is invasive and needs hardware implantation. Beyond conventional rTMS, emerging non-invasive neuromodulation techniques are being explored for AUD. These include theta-burst stimulation (TBS), deep TMS, and transcranial direct current stimulation (tDCS) [4–6]. While these approaches offer promise, their efficacy in achieving sustained abstinence in severe, treatment-refractory AUD remains under investigation, and similar to rTMS, their ability to directly modulate deep subcortical structures like the NAc is inherently limited.

Transcranial-focused ultrasound (tFUS) has risen as a novel non-invasive brain stimulation technique. Low-intensity tFUS (LIFU)delivers non-thermal, focused acoustic pressure waves to specific subregions of the brain through the skull [7]. Recent studies showed the effectiveness of NAc LIFU for substance use disorder [8, 9]. Magnetic resonance-guided focused ultrasound surgery (MRgFUS) as a minimally invasive thermal injury technique represents a novel treatment option for various psychiatric disorders, such as obsessive-compulsive disorder (OCD) and major depressive disorder (MDD) [10, 11]. The ability to precisely ablate the brain areas offers a paradigm-shifting approach to disrupt addiction-related circuitry while avoiding complications of invasive surgery. However, its application in AUD remains exploratory.

As a primary input structure of the basal ganglia limbic loop, the nucleus accumbens (NAc) integrates information from the frontal cortex and mesolimbic structures to mediate motivational, rewarding, and emotional processes [12]. As a result, the NAc has been implicated in numerous neurological and psychiatric disorders, and it is the target of the effects of certain psychoactive drugs and some potentially therapeutic devices [12]. Previous studies have shown that DBS in the NAc area substantially reduces alcohol consumption and cocaine use, attenuates smoking dependency, and promotes sustained heroin abstinence [13–16].

Here, we present the first known report of the treatment of AUD by bilateral NAc MRgFUS in a 54-year-old male with a 30-year history of severe AUD, resistant to multiple lines of therapy. We hypothesize that NAc modulation via MRgFUS may attenuate alcohol craving by normalizing reward system hyperactivity, offering a novel therapeutic avenue for treatment-refractory AUD.

## Case presentation

### History

A 54-year-old married man with a longstanding history of alcohol consumption dating back to adolescence. The individual had a business failure approximately 30 years prior that resulted in severe anxiety and depression, after which he developed AUD, which was diagnosed independently by two experienced psychiatrists based on the Diagnostic and Statistical Manual of Mental Disorders-Fifth Edition (DSM-5) criteria [17]. He did not receive any treatment until 2005, when he drank for more than 10 days at a time, as long as he started drinking. He had a history of alcohol intoxication and had severe withdrawal reactions, including leg weakness and hand tremors, during the period when he stopped drinking. Since 2005, he has undergone approximately 50 compulsory hospitalizations for abstinence (longest > 1 month, shortest 3–4 days), receiving primarily pharmacological treatment. He had just been discharged from the hospital for a week at the time of the preoperative psychological evaluation, when his depression and anxiety levels were low (Table 1). Three months before the surgery, he crashed his car into a tree due to drunk driving. His pre-operative medication regimen Paroxetine (20 mg/day in the morning) and Quetiapine (200 mg/day at night) had been stabilized and maintained at these doses for more than ten years prior to the MRgFUS surgery. The Sodium Valproate (1000 mg/day, administered as 500 mg twice daily) had been taken by three months before the surgery. At the last follow-up, the drugs used were Paroxetine (20 mg/d), Topiramate(200 mg/d), and Quetiapine (200 mg/d).

**Table 1:** Clinical outcome measures

Scales	Baseline	Follow-up			
		1 month	3 months	9 months	12 months
MoCA	27	/	/	/	27
HAMD-17	10	3	4	2	4
HAMA-14	8	7	7	3	3
BDI-II	21	2	2	1	2
BAI	5	3	3	3	2
VAS	6	0	0	0	0
SADQ-C	22	/	/	/	6
MAST	15	/	/	/	0
PSQI	14	11	11	10	10
SF-36					
Total score	433.56	/	780	/	814
Physical functioning	85	/	95	/	95
Role-physical	25	/	100	/	100
Body pain	41	/	62	/	84
General health	47	/	52	/	60
Vitality	70	/	95	/	95
Social functioning	55.56	/	100	/	100
Role-emotional	0	/	100	/	100
Mental health	60	/	76	/	80
Reported health transition	50	/	100	/	100
WHOQOL-BREF					
Physical health	39.29	/	60.71	/	67.86
Psychological	54.17	/	83.33	/	83.33
Social relationship	50	/	75	/	75
Environment	68.75	/	75	/	75

Note: Scale ranges and clinical thresholds: Scale ranges and clinical thresholds: BAI (0–7 normal, 8–15 mild anxiety, 16–25 moderate anxiety, 26–63 severe anxiety); BDI-II (0–13 normal, 14–19 mild depression, 20–28 moderate depression, 29–63 severe depression); HAMD-17 (＜8 normal, 8–17 mild depression, 18–24 moderate depression, and ＞24 severe depression); HAMA-14 (＜7 normal, 7–13, mild anxiety; 14–20, moderate anxiety; 21–28, significant anxiety; and > 28, severe anxiety); VAS (0 = no craving, 10 = most intense craving). For MAST, SADQ-C, and PSQI, higher scores indicate greater severity. For SF-36 and WHOQOL-BREF, higher scores indicate better status/functioning. BAI, Beck Anxiety Inventory; BDI-II, Beck Depression Inventory-II; HAMD-17, 17-item Hamilton Depression Rating Scale; HAMA-14, 14-item Hamilton Anxiety Rating Scale; MAST, Michigan Alcoholism Screening Test; PSQI, Pittsburgh Sleep Quality Index; SADQ-C, Severity of Alcohol Dependence Questionnaire-Community; SDC, Sheehan Disability Scale; SF-36, 36-Item Short Form Survey; VAS, Visual Analogue Scale

### Clinical assessment

The clinical outcomes were obtained before surgery (baseline), 1 month, 3 months, 9 months, and 12 months follow-up. Craving for alcohol was evaluated using a Visual Analog Scale (VAS, 0–10), chosen for its simplicity and sensitivity in tracking rapid changes in subjective craving. Abstinence was assessed through patient self-report and corroborated by family members during follow-up interviews. Depression and anxiety were evaluated by an experienced psychiatrist by using the 17-item Hamilton Depression Scale (HAMD-17) [18] and 14-item Hamilton Anxiety Scale (HAMA-14) [19]. We also obtained self-report measures of depression, anxiety, alcoholism screening, alcohol dependence, sleep quality, health status by using the Beck Depression Inventory (BDI-II) [20], Beck Anxiety Inventory (BAI) [21], Michigan Alcoholism Screening Test (MAST) [22], Severity of Alcohol Dependence Questionnaire-Community (SADQ-C) [23], Pittsburgh Sleep Quality Index (PSQI) [24], 36-Item Short Form Health Survey (SF-36) [25], respectively.

The assessment schedule was designed to balance comprehensiveness with patient burden. Primary dynamic outcomes (craving, mood, sleep) were measured at all time points. The MoCA, SADQ-C, and MAST were administered at baseline and 12-month endpoint as they serve as summary measures of global cognitive status and dependence severity. The full SF-36 and WHOQOL-BREF were administered at baseline, 3 months, and 12 months due to their length, with the 3-month time point chosen to capture early changes in quality of life.

### MRgFUS procedure

**Fig. 1:**
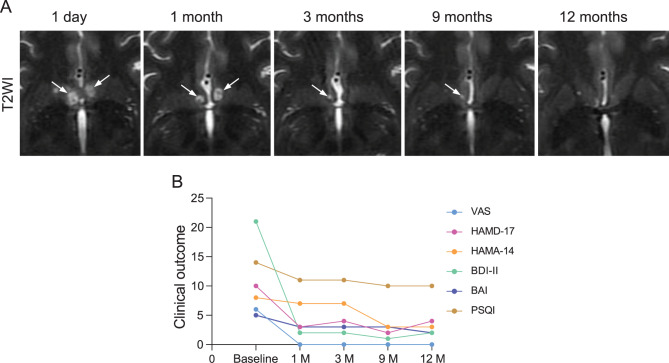
Lesion location and clinical outcome following bilateral nucleus accumbens MRgFUS ablation. (A) the size of the bilateral NAc lesion after MRgFUS. White arrows point to the lead NAc lesion area. T2WI, T2-weighted image. (B) longitudinal changes in clinical outcome measures. The line graph depicts scores from baseline through the 12-month follow-up for key clinical scales: visual analog Scale (vas) for craving, 17-item Hamilton depression rating Scale (HAMD-17), 14-item Hamilton anxiety rating Scale (HAMA-14), Beck depression Inventory-II (BDI-II), Beck anxiety Inventory (BAI), and Pittsburgh sleep quality index (PSQI). Lower scores on all scales indicate improvement. The sustained reduction in vas to zero correlates with complete abstinence, while parallel improvements in mood and sleep scales suggest broader therapeutic benefits. M, month

The patient’s skull density ratio (SDR) was calculated from the preoperative CT scan as 0.54, which meets the clinical threshold for effective ultrasound penetration through the cranium. The technique for this procedure has been described previously. Briefly, high-resolution 3D T1-weighted images were acquired using a 3.0T MRI scanner (Discovery 750W, GE healthcare) (sequence parameters: TR = 7.4 ms, TE = 1.0 ms, slice thickness = 1 mm). Data were imported into stereotactic planning software (Exablate Neuro 4000) for bilateral NAc targeting. The NAc targets were localized using stereotactic coordinates relative to the anterior commissure-posterior commissure (AC-PC) line: X-axis (mediolateral): 4.5–5 mm lateral to the midline; Y-axis (anteroposterior): 3.5–5.5 mm anterior to the anterior commissure (AC); Z-axis (dorsoventral): 1–5 mm inferior to the AC-PC line. After scalp shaving, a stereotactic head frame (CRW frame) was mounted and coupled to the ExAblate Neuro system (InSightec, Haifa, Israel), which features a hemispherical phased-array transducer (1,024 elements, central frequency 650 kHz). Subtherapeutic sonications (power ≤ 500 W, duration 20 s) were applied to confirm targeting accuracy. Calibration Protocol: Prior to formal treatment, four sonications were performed with three-plane alignment (axial/coronal/sagittal). High-intensity ultrasound energy was delivered only after confirming no spatial deviation in all planes. The patient was provided with a handheld pause button to terminate treatment immediately if discomfort occurred during any sonication. Real-time MR thermometry (proton resonance frequency method) ensured focal temperatures ≤ 43 °C to avoid unintended tissue damage. Acoustic power was incrementally increased (range: 900–1200 W) until the target region reached 51–55 °C for ≥ 3 seconds. The number of active transducer elements during treatment was 957, with a total skull surface area of 417 cm^2^ irradiated. Thermal lesion formation was monitored via real-time T2-weighted sequence. Each NAc received two sonications, with a total procedure duration of 4 hours. Post each sonication, nursing staff conducted immediate assessments of the patient’s chief complaints. Antiemetic drugs were administered promptly for symptomatic management of nausea or related adverse reactions. Postoperative contrast-enhanced T1 and T2-FLAIR sequences were acquired within 24 hours to confirm the ablation zone location and extent (Fig. 1A). Additionally, a 9-min resting-state functional MRI (2D gradient-echo EPI sequence) scan was acquired during each evaluation visit: 180 volumes, TR = 3 s, TE = 30 ms, flip angle = 90°, voxel size = 3 mm isotropic. Acute postoperative reactions (e.g., headache, vertigo) were assessed by a multidisciplinary team (neurosurgeon and psychiatrist) at 1 week, 1 month, 3 months, 9 months, and annually thereafter.

### MRI image processing

Region-of-interest (ROI)-wise functional connectivity (FC) changes were also explored. Functional MRI data were preprocessed using *fMRIPrep* (docker version 24.1.1) [26], and subsequent denoising and computation of FC map were carried out using *XCP-D* (docker version 0.10.7) [27]. FC matrices were derived using the 4S156 parcellation atlas and compared to the GSP1000 normative functional connectome - a resting-state fMRI dataset comprising 1,000 healthy individuals from the Genomic Superstruct Project [28]. For structural and functional lesion-based analysis, the bilateral MRgFUS lesions were segmented on T2-weighted images obtained at the 1-month follow-up, and the left-sided lesion was non-linearly flipped to the right side. Functional and structural connectivity analyses were performed using the GSP1000 normative connectome and MGH-USC HCP normative connectome in the Lead Connectome Mapper toolbox (Leadbs toolbox v3.2, Matlab R2023a), respectively [29, 30].

## Results

At the baseline assessment, the weighted and simple total scores of MAST were 35 and 15, respectively. After the surgery, the patient’s VAS for alcohol decreased from 6 to 0, and during the entire research period (12 months), he reported no alcohol cravings and had not consumed alcohol (Table 1, Fig. 1B). During a one-month follow-up, the HAMD-17 and HAMA-14 scores decreased from 10 to 3 and 8 to 7, respectively (Table 1). A self-rating scale for depression (BDI-II) and anxiety (BAI) scores decreased from 21 to 2 and from 5 to 3, respectively. The Montreal Cognitive Assessment (MoCA) score was 27 both before the operation and at the 12-month follow-up. Two summary measures of the SF-36 indicated that his physical component score (PCS) increased from 198 to 339, and his mental component score (MCS) increased from 185.56 to 375 at the 12-month follow-up. His difficulty falling asleep and waking up at night improved.

In addition to the improvement in the scores of the relevant scales mentioned above, this patient expressed satisfaction with the treatment because he no longer had a strong desire to drink alcohol and was no longer repeatedly hospitalized for quitting drinking. As a result, he was able to devote himself wholeheartedly to his work, which he had been unable to do for many years.

At both 1 and 3-month follow-ups, the patient reported a loss of desire for many things in life, such as delicious food. Upon further review of our clinical notes, the patient spontaneously reported the return of his normal desire for food and other pleasures during a routine check-in call at the 6-month post-operative mark. This was subsequently confirmed to have been sustained at the formal 9-month follow-up visit. This surgery did not induce hypomania or other significant adverse side effects.

**Fig. 2:**
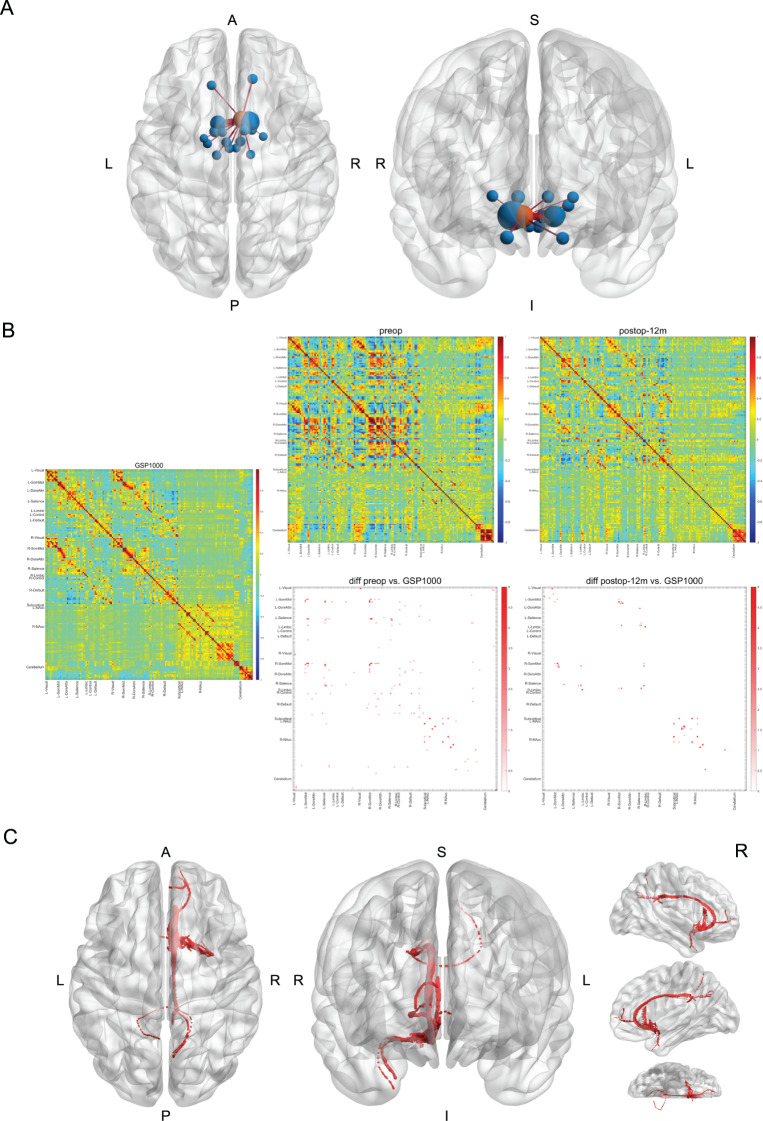
The MRgFUS lesion-based functional connectivity (FC) (A), whole brain FC correlation coefficient R-matrix (B), and structural connectivity (C). The 4S156 atlas was used for cortical and subcortical parcellation. The MRgFUS lesion (red round) was segmented based on the T2-weighted image of the 1-month follow-up. For visualization purposes, only parcellations (blue rounds) having a Pearson’s correlation coefficient > 0.1 with the lesion were shown (**b**). (**c**) The Pearson correlation coefficient (**r**) Was computed for each pair of brain parcellations. The resulting R-matrix was then transformed using Fisher’s z-transformation before visualization. Each edge’s *r* value was compared against the normative connectome derived from the GSP1000 dataset using the Crawford and Howell single-case t-test. Multiple comparisons were corrected using the false discovery rate (FDR) method. (D) The MRgFUS lesion was segmented based on the T2-weighted image of the 1-month follow-up. The MGH-USC HCP normative connectome was used for structural connectivity mapping

Lesion-based functional connectivity analysis revealed a connection between the lesion and several subcortical and cortical regions, including the contralateral NAc, bilateral somatosensory thalamus, orbitofrontal cortex, amygdala, hypothalamus, subthalamic nucleus, and mamillary nucleus (Fig. 2A, supplementary Table 1–2). At the 12-month follow-up, functional connectivity patterns partially ‘normalized’ relative to the GSP1000 normative connectome (Fig. 2B). The lesion-based structural connectivity mapping demonstrated predominant connections between the lesion and ipsilateral cingulate cortex, as well as frontal and temporal lobes (Fig. 2C).

## Discussion and conclusions

MRgFUS is a non-invasive technique that offers several advantages, including non-invasiveness, no need for general anesthesia, real-time target localization, and real-time temperature monitoring [31]. This is the first report to evaluate the long-term efficacy and safety of bilateral NAc ablation using MRgFUS in a patient with treatment-refractory AUD. In this study, the patient diagnosed with AUD for 30 years had no craving for alcohol since NAc MRgFUS surgery, and this has been sustained until the last follow-up (12 months). Besides, the results showed sustained and long-term improvements in depression, quality of life, and social functioning in this case.

NAc is a pivotal node within the mesolimbic dopamine reward pathway and drives reinforcement learning and craving in addiction [12]. MRgFUS targeting NAc to reduce alcohol craving may function by modulating activity in the midbrain limbic circuit, which includes dopaminergic pathways. A preclinical study found that LIFU modulates dopamine in a mesolimbic reward circuit [32]. Delivering low-frequency TUS to the NAc can modulate brain activations, functional connectivity within the reward network, and affect reward-related behaviours [33, 34]. In this case report, the resting-state functional connectivity findings offer a potential systems-level mechanism for the sustained reduction in craving and abstinence observed in this patient. The post-ablation ‘normalization’ of functional connectivity patterns, as measured against the normative connectome, suggests that the therapeutic effect of NAc ablation may arise from a fundamental restructuring of addiction-related networks.

Compared to invasive DBS, MRgFUS eliminates risks of hemorrhage, infection, and hardware complications. Its real-time MRI thermometry ensures millimeter-level precision in targeting the NAc, avoiding adjacent structures. Unlike rTMS, which fails to reach deep subcortical targets, MRgFUS directly engages addiction-related nuclei, offering a unique advantage for AUD treatment.

The application of ablative MRgFUS to the NAc for AUD necessitates careful ethical deliberation. While the procedure is “incision-less” and avoids the immediate risks of open surgery, it results in permanent, irreversible tissue destruction. This stands in stark contrast to neuromodulation approaches like deep brain stimulation (DBS) or low-intensity focused ultrasound (LIFU), which are adjustable and reversible. The transient loss of desire for pleasurable activities, such as enjoying food, reported at the 1- and 3-month follow-ups, is a clinically significant observation. The NAc is a well-established hub for encoding motivation and reward salience for both natural reinforcers (e.g., food, social interaction) and drugs of abuse [35, 36]. The ablation of this region likely transiently dampened this circuit, thereby reducing the motivational drive for alcohol—the intended therapeutic effect—but also concomitantly blunted the experience of pleasure from other sources. The resolution of these symptoms by the 6-month mark suggests a capacity for functional reorganization or compensatory mechanisms within the distributed reward network over time. Nevertheless, this phenomenon underscores the profound ethical considerations of irreversibly lesioning a key node of the reward system. While the symptom resolved and the benefit of eliminating life-threatening AUD in this treatment-refractory individual outweighed the temporary risk, this observation warrants extreme caution. It highlights the potential for this intervention to alter fundamental aspects of human experience and motivation. Consequently, this intervention must be reserved for a highly select group of patients with severe, treatment-refractory illness, where the debilitating burden of their addiction clearly outweighs the potential risks. A comprehensive and rigorous informed consent process is paramount, ensuring patients fully comprehend the experimental nature, the irreversibility of the lesion, the spectrum of potential adverse effects, and the unknown long-term consequences.

## Limitations

This report has several limitations. First, as a single-case report lacking control groups, blinding, or randomization prevents definitive causal inference. Confounding factors, including concurrent pharmacotherapy and recent hospitalization, cannot be discounted. Furthermore, the lack of blinding introduces the potential for placebo/nocebo effects and observer bias, while the single-case design cannot rule out regression to the mean as an explanation for the outcomes. Second, the absence of objective biomarkers and verification measures. Abstinence was primarily confirmed through self-report and corroborated by family members, lacking objective biological confirmation such as phosphatidylethanol (PEth), blood alcohol levels, or urine toxicology screens. Furthermore, no longitudinal biological tests, such as liver enzymes (AST, ALT), were performed, which could have provided objective evidence of physiological recovery following sustained abstinence. Similarly, reported improvements in sleep were assessed using the subjective PSQI rather than objective measures like actigraphy. The implementation of these robust, quantitative verification tools is a crucial prerequisite for future controlled trials to unequivocally confirm the efficacy, safety, and physiological benefits observed in this case. Third, the assessment of craving relied on the unidimensional VAS, which, while clinically useful, is not as comprehensive as multi-dimensional instruments for capturing the complexity of craving.

Our findings in this single case demonstrate that bilateral NAc ablation via MRgFUS may represent a feasible investigational approach for severe, treatment-refractory AUD. The sustained elimination of craving and abstinence over 12 months, alongside improvements in mood, sleep, and quality of life, suggests a potential clinical benefit in this patient. The procedure was well-tolerated without serious adverse events, supporting its preliminary safety profile in this context. This case provides preliminary proof-of-concept that MRgFUS ablation of the NAc can durably disrupt addictive behaviors in a single individual while preserving global cognitive function. It positions this technique as a candidate worthy of further rigorous investigation in controlled clinical trials. Future multi-center, blinded, and randomized studies with larger samples, objective biomarkers, and long-term monitoring are essential to validate its efficacy, safety, and place in the therapeutic arsenal for AUD.

## Electronic supplementary material

Below is the link to the electronic supplementary material.

Supplementary Material 1

## Data Availability

The data that support the findings of this study are available on request from the corresponding author. The data are not publicly available due to privacy or ethical restrictions.

## Abbreviations

AUD: Alcohol use disorder
BAI: Beck Anxiety Inventory
BDI: Beck Depression Inventory
DBS: Deep brain stimulation
FC: Functional connectivity
HAMA-14: 14-item Hamilton Anxiety Scale
HAMD-17: 17-item Hamilton Depression Scale
MDD: Major depressive disorder
MRgFUS: Magnetic resonance-guided focused ultrasound
NAc: Nucleus accumbens
rTMS: Repeated transcranial magnetic stimulation
SF-36: 36-Item Short Form Health Survey
VAS: Visual analog scale
